# Dendrometric traits for developing biomass equations of tree and shrub species in carbon sequestration projects

**DOI:** 10.3897/BDJ.13.e164624

**Published:** 2025-10-21

**Authors:** Chun-Jing Wang, Xue Wang, Mei-Quan Wang, De-Chao Chen, Wu-Xian Yan, Dong-Zhou Deng

**Affiliations:** 1 Ecological Conservation, Restoration and Resource Utilization on Forest and Wetland Key Laboratory of Sichuan Province, Sichuan Academy of Forestry, Chengdu, China Ecological Conservation, Restoration and Resource Utilization on Forest and Wetland Key Laboratory of Sichuan Province, Sichuan Academy of Forestry Chengdu China; 2 Wolong Forest Ecology Observation and Research Station of Sichuan Province, Sichuan Aba, China Wolong Forest Ecology Observation and Research Station of Sichuan Province Sichuan Aba China; 3 Sichuan Provincial Forestry and Grassland Key Laboratory of Combating Desertification, Chengdu, China Sichuan Provincial Forestry and Grassland Key Laboratory of Combating Desertification Chengdu China

**Keywords:** base perimeter, biodiversity, canopy breadth, China, forest management, height, woody species

## Abstract

**Background:**

The advancement of carbon sequestration projects holds significant potential to deliver mutually beneficial outcomes for both the environment and the economy. In this context, biomass models have been extensively developed to estimate the aboveground biomass of woody plants — such as trees and shrubs — using dendrometric characteristics, like diameter and height. The datasets presented in this study compile dendrometric traits from multiple tree and shrub species, supporting the construction of robust biomass models. As a result, tree and shrub biomass can serve as integral indicators for evaluating the effectiveness of carbon sequestration projects, incorporating key factors such as diameter, height and plantation density. By establishing reliable biomass estimation models, it becomes possible to enhance the monitoring and verification of carbon storage, thereby providing a scientific basis for the planning, management and policy-making of carbon sink initiatives. This approach contributes significantly to ecological restoration and climate change mitigation efforts.

**New information:**

This study presents two dendrometric datasets of individual trees and shrub bushes from carbon sequestration projects in north-western China, covering sites in Xining and Haidong (Qinghai Province), Tianshui (Gansu Province) and Aba (Sichuan Province). Specifically, the tree dataset comprises measurements of canopy breadth (in two perpendicular directions), height, diameter at breast height (DBH) and base perimeter for 2084 individuals across 25 species. The shrub dataset includes crown diameter (in two perpendicular directions), height and basal perimeter for 998 bushes across 36 species. These dendrometric traits serve as key parameters in biomass estimation equations. Furthermore, as the diameter and height of trees and shrubs significantly influence understorey plant diversity — primarily through their effects on stand density, species interactions and community composition — these datasets are valuable for advancing biomass modelling and assessing plant diversity outcomes under conservation management.

## Introduction

The development of forest sinks could play a major role in the effort to slow the accumulation of atmospheric carbon dioxide, as well as in energy conservation and emission reduction in industrial production (Pugh et al. 2019, Paul and Roxburgh 2020). However, the long-term stressful utilisation of forests has led to ecosystem degradation and carbon loss (Gao et al. 2020). In this context, a series of forest projects have been developed, including the plantation of marginal agricultural land, conservation reserve programmes, afforestation of agricultural land and forest restoration projects for carbon sequestration (Brack and King 2021, Oldfield et al. 2022). The development of carbon sequestration projects in plantation forests has the potential to provide mutually beneficial outcomes for the environment and economy (Brack and King 2021, Pan et al. 2024, Searchinger et al. 2022, Wang et al. 2025). These projects have made large contributions to addressing climate change, supporting local communities and smallholders and conserving biodiversity (Brack and King 2021, Pan et al. 2024, Pugh et al. 2019). Tree and shrub biomass play an important role in carbon sequestration project development (Pugh et al. 2019, Yadav et al. 2022).

Tree and shrub biomass can shape plant diversity and ecosystem functions across different spatial scales from community to global scales (Gao et al. 2020, Chen et al. 2018, Zhang et al. 2024, Godlee et al. 2021). Plant community diversity of forest understorey is closely related to tree and shrub biomass in forests (Wei et al. 2021, Wan et al. 2025). Understanding tree and shrub biomass helps contribute to exploration on forest carbon cycling and implementing forest carbon offset activities (Pugh et al. 2019, Gao et al. 2020, Zhang et al. 2024, Brack and King 2021, Godlee et al. 2021). Biomass equations of tree and shrub biomass are commonly used widely in forest management and ecological restoration (Luo et al. 2020, Zhang et al. 2024, Wang et al. 2021,Wang et al. 2024). Luo et al. (2020) provided a review of biomass equations for Chinese tree species, highlighting that dendrometric traits are effective predictor variables of biomass equations for tree species. Tree biomass equations refer to quantitative relationships between tree biomass and dendrometric traits, for example, tree diameter and height. The comprehensive database of dendrometric traits of tree individuals is critical to the assessment of tree biomass in forests.

Wang et al. (2021) provided a biomass equation dataset for common shrub species in China, based on the available literature (journals, books and postgraduate dissertations) between 1982 and 2019 from several comprehensive online literature databases. Shrub biomass equations refer to quantitative relationships between the biomass of the whole individual or different components (such as stems, branches, leaves and roots) and one or several dendrometric variables (such as shrub height, basal diameter and crown projection). We usually run the shrub biomass equations using the dendrometric traits, for example, height, crown diameter and basal diameter. However, it is needed to obtain detailed data on dendrometric variables for biomass equations of tree and shrub species.

Here, we provided two datasets of dendrometric traits on tree individuals and shrub bushes, respectively, in carbon sequestration projects of western China. The dendrometric traits of these two datasets refer to the parameters of tree and shrub biomass equations of Luo et al. (2020) and Wang et al. (2021). The data can improve the accuracy of biomass equations of tree and shrub species. Effective assessment of tree biomass performance can guide the economic and equipment investment decisions for plantation forests by policy-makers and carbon emissions trading for social development.

## General description

### Purpose

Our study provided the key parameters of biomass equations for tree and shrub species. The studies of Luo et al. (2020) and Wang et al. (2021) contribute to the development of tree and shrub biomass equations reported in diverse types of literature (e.g. journals, books and reports). However, the fieldwork data should be collected for calculating biomass and correcting the biomass equations. Although the database of our study was restricted to carbon sequestration projects of Xining and Haidong, Qinghai Province Tianshui, Gansu Province and and Aba, Sichuan Province, China, most of the planted tree and shrub species were covered by fieldwork. This provides first-hand data for assessing the woody plant biomass of western China and developing the robust biomass equations for tree and shrub species.

## Project description

### Study area description

The study areas of our study included Xining, Haidong and Tianshui cities and Aba Tibetan and Qiang Autonomous Prefecture in western China. Xining and Haidong are located in the Yellow River–Huangshui River Valley in Qinghai Province, China at altitudes of 1700 m to 3800 m (Ma et al. 2024, Wang et al. 2023). Qinghai Province is the north-eastern part of the Qinghai–Tibet Plateau, far inland (Ma et al. 2024, Wang et al. 2023). It is not only an important part of the Qinghai–Tibet Plateau, but also the most representative region in terms of the area’s biodiversity. The study area is characterised by the plateau arid and semi-arid continental climate. The annual average precipitation is 381.1 mm and the annual average temperature ranged from 3.1ºC to 7.9ºC (Ma et al. 2024, Wang et al. 2025). Tianshui is located between 34°36′–35°42′ N and 105°32′–106°56′E, with a total area of approximately 14000 km^2^ in Gansu Province. Tianshui is a typical arid inland climate zone. The climate of Tianshui can be divided into temperate continental arid and temperate semi-humid regions (Hui et al. 2011, Wang et al. 2025). The climate of Aba Prefecture is predominantly characterised by a highland monsoon pattern. It features severe continental conditions with pronounced seasonal variations. Temperature and precipitation levels are highly contingent on altitude, creating a complex spectrum of local microclimates.

### Design description

**Figure 1.**
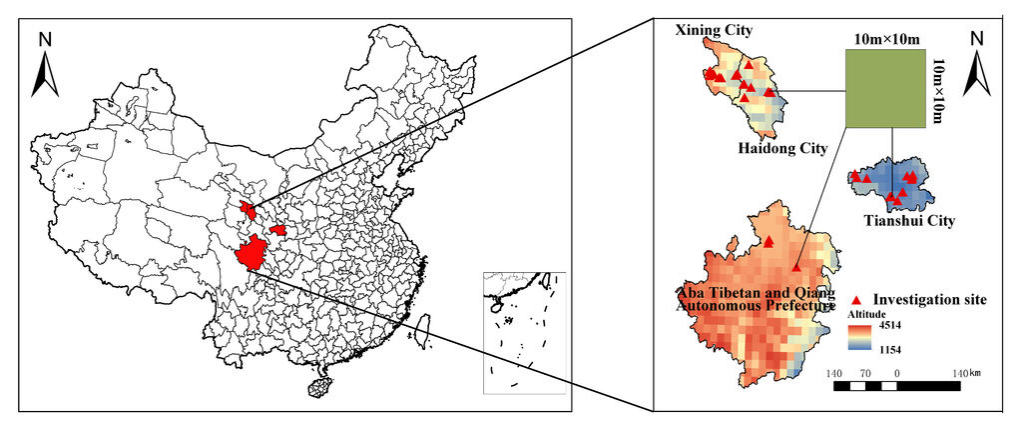
Investigation sites (10 × 10 m^2^) in our study.

We investigated different study areas with carbon sequestration projects, each defined by a circle with a 10-km radius, to assess plant species diversity in plantation forests managed for carbon sequestration projects. These study areas have similar environmental conditions (such as soil and climate) and cover the full altitude range of Xining, Haidong, Tianshui cities and Aba Tibetan and Qiang Autonomous Prefecture in western China (Hui et al. 2011, Ma et al. 2024). Five sites were investigated in each study area. The total number of sites was 140, as shown in Fig. 1. The selected habitat types were intended to be relatively frequent in the study area (i.e. very rare and extreme habitats were avoided). One to three typical target habitats were sampled as pairs of plots, each one under different anthropogenic disturbance conditions (relatively natural vs. disturbed) within each study area. The terrestrial plant communities were established in plantation forests managed for carbon sequestration projects along an altitudinal gradient from June 2022 to August 2025. Following the methods described by Fang et al. (2009), we recorded the following data:

1. geographic coordinates (latitude and longitude) of the centre of the study sites;
2. altitudes of the centre of the study sites;
3. abundance and coverage of plant species within the study site; and
4. vascular plant species list following the Plant List.

However, we excluded the data on longitude and latitude due to the Law of the People's Republic of China on Guarding State Secrets. These variables could not affect the development of tree and shrub biomass equations and the estimation of carbon sequestration projects in Xining, Haidong and Tianshui cities and Aba Prefecture in western China.

## Sampling methods

### Sampling description

We conducted the investigations of tree individuals. For each investigation site, we measured canopy breadth from two vertical directions for tree individuals. We selected two opposing measurement points at the widest point of the tree crown, ensuring that these two points are located at the widest points in the north-south and east-west directions of the tree crown, respectively. Then, we used a tape to measure the distance between these two measuring points and record the width in the north-south and east-west directions, respectively. Thus, we obtained two data groups of canopy breadth for each species individual. For short individuals, we used a ruler to measure the height. For tall individuals, we used a long ruler and stood far enough away from the individual and estimated the height of the tree individual by the angle between the ruler and the top of the tree individual. By utilizing the trigonometric functions, we measured the distance between us and the tree base, as well as the angle between ourselves and the tree tip and then calculated the height of the tree individuals. We measured basal diameter and diameter at breast height (i.e. 1.3 m above the soil surface) using a ruler.

We conducted similar investigations for shrub bushes. For each investigation site, we measured crown diameters from two vertical directions for shrub bushes (i.e. aggregate measurements of entire clumps) in each bush. We selected two opposing measurement points at the widest point of the shrub crown, ensuring that these two points are located at the widest points in the north-south and east-west directions of the shrub crown in each bush, respectively. Then, a tape was used to measure the distance between these two measuring points and to record the width in the north-south and east-west directions, respectively, for each bush. We collected two data groups of shrub crown diameters for each bush. Then, we used a ruler to measure the maximum height amongst the bushes directly. We used a tape to measure the basal perimeter of each bush, closely associated with the basal diameter of the shrub bush.

## Geographic coverage

### Description

The investigation sites are located between 26.68°–36.95°N and 101.06°–106.19°E (Fig. 1).

### Coordinates

26.68°N and 36.95°N Latitude; 101.06°E and 106.19°E Longitude.

## Taxonomic coverage

### Description

The 61 taxa including 36 shrubs and 25 trees represented in the dataset. Specifically, these plant species belong to a diverse genus, namely, 19 and 24 genera for trees and shrubs, respectively.

### Taxa included

**Table:** 

Rank	Scientific Name	
species	*Amorpha fruticosa* Linnaeus, 1753	
species	*Aralia elata* (Miq.) Seemann, 1868	
species	*Berberis thunbergii* DC., 1821	
species	*Berberis vernae* Schneid., 1913	
species	*Caragana korshinskii* Komarov, 1909	
species	*Caragana tibetica* Komarov, 1909	
species	*Cotoneaster multiflorus* Bunge, 1830	
species	*Dasiphora glabra* (G. Lodd.) Soják, 1983	
species	*Dasiphora fruticosa* (L.) Rydb.,1898	
species	*Daslphora parvifolla* (Fisch.) Juzep, 1941	
species	*Elaeagnus pungens* Thunberg, 1784	
species	*Elaeagnus umbellata* Thunberg, 1784	
species	Hippophae rhamnoides Linn. subsp. sinensis Rousi, 1971	
species	*Hippophae tibetana* Schltdl.,1863	
species	*Indigofera tinctoria* Linnaeus, 1753	
species	*Lonicera hispida* Pall. ex Roem. & Schult., 1819	
species	*Lonicera japonica* Thunberg, 1784	
species	*Lonicera rupicola* Hook. f. & Thomson,1858	
species	Lonicera rupicola var. syringantha (Maxim.) Zabel, 1903	
species	*Myricaria paniculata* P. Y. Zhang, 1984	
species	*Periploca sepium* Bunge, 1835	
species	*Prunus triloba* Lindley, 1857	
species	*Rhamnus utilis* Decaisne, 1857	
species	*Rhus typhina* Linnaeus, 1756	
species	*Ribes himalense* Royle ex Decne., 1844	
species	*Rosa xanthina* Lindley, 1820	
species	*Salix oritrepha* C. K. Schneid., 1916	
species	*Salix sinica* (Hao) C. Wang et C. F. Fang, 1936	
species	*Salix taoensis* Goerz ex Rehder & Kobuski, 1932	
species	*Sibiraea angustata* (Rehder) Hand.-Mazz., 1933	
species	*Sophora davidii* Komarov ex Pavolini, 1908	
species	*Sorbaria sorbifolia* (L.) A. Braun, 1864	
species	*Spiraea alpina* Pallas, 1784	
species	*Syringa oblata* Lindley, 1859	
species	Syringa reticulata subsp. amurensis (Rupr.) P. S. Green & M. C. Chang, 1983	
species	*Viburnum rhytidophyllum* Hemsley, 1888	
species	*Adinandra millettii* (Hook. & Arn.) Benth. & Hook. f. ex Hance, 1878	
species	*Ailanthus altissima* (Mill.) Swingle, 1916	
species	*Crataegus pinnatifida* Bunge, 1835	
species	*Elaeagnus angustifolia* Linnaeus, 1753	
species	*Juglans regia* Linnaeus, 1753	
species	*Juniperus chinensis* Roxburgh, 1832	
species	*Juniperus formosana* Hayata, 1908	
species	*Koelreuteria paniculata* Laxmann, 1772	
species	*Larix gmelinii* (Rupr.) Kuzeneva, 1920	
species	*Picea crassifolia* Komarov, 1923	
species	*Pinus tabuliformis* Carrière, 1867	
species	*Platycladus orientalis* (L.) Franco, 1949	
species	Populus alba var. pyramidalis Bunge, 1854	
species	*Populus davidiana* Dode, 1905	
species	*Populus simonii* Carrière, 1867	
species	*Prunus armeniaca* Linnaeus, 1753	
species	*Prunus cerasifera* Ehrhart, 1784	
species	*Prunus pseudocerasus* Lindl., 1830	
species	*Prunus sibirica* Linnaeus, 1753	
species	*Pyrus betulifolia* Bunge, 1835	
species	*Robinia pseudoacacia* Linnaeus, 1753	
species	*Salix matsudana* Koidz., 1915	
species	*Ulmus pumila* Linnaeus, 1753	
species	*Zanthoxylum bungeanum* Maximowicz, 1871	
species	*Zelkova serrata* (Thunb.) Makino, 1903	

## Temporal coverage

**Data range:** 2022-6-01 – 2025-8-01.

## Usage licence

### Usage licence

Other

### IP rights notes

CC-BY 4.0

## Data resources

### Data package title

Dendrometric traits of biomass equations for tree and shrub species in carbon sequestration projects of north-western China

### Resource link

https://doi.org/10.57760/sciencedb.20996

### Number of data sets

2

### Data set 1.

#### Data set name

Tree

#### Description

Dendrometric traits of biomass equations for tree species in carbon sequestration projects of north-western China.

**Data set 1.** 

Column label	Column description
Species	The full scientific name of the plant species.
Family	The full scientific name of the plant family.
Genus	The full scientific name of the plant genus.
Height (m)	The height of tree species recorded for each individual. Unit: m.
Diameter at breast height (cm)	The diameter at breast height. The DBH of tree species recorded for each individual. Unit: cm.
Base diameter (cm)	The base diameter of tree species recorded for each individual. Unit: cm.
Canopy breadth1 (cm)	The canopy breadth of tree species for each individual with the first record. Unit: cm.
Canopy breadth2 (cm)	The canopy breadth of tree species for each individual with the second record. Unit: cm.
Branch	The branch or not.

### Data set 2.

#### Data set name

Shrub

#### Description

Dendrometric traits of biomass equations for shrub species in carbon sequestration projects of north-western China.

**Data set 2.** 

Column label	Column description
Species	The full scientific name of the plant species.
Famliy	The full scientific name of the plant family.
Genus	The full scientific name of the plant genus.
Maximum height (m)	The maximum height of shrub species recorded amongst different individuals in each bush. Unit: m.
Individual number	The individual number of shrub species recorded in each bush. No unit.
Base diameter (cm)	The base diameter of shrub species recorded amongst different individuals together in each bush. Unit: cm.
Crown diameter1 (cm)	The crown diameter of shrub species recorded amongst different individuals together in each bush with the first measure. Unit: cm.
Crown diameter2 (cm)	The crown diameter of shrub species recorded amongst different individuals together in each bush with the second measure. Unit: cm.

## Additional information

### General observations

We recorded tree heights ranging from 0.2 m to 18.7 m, with mean values across species varying between 0.63 m and 7.51 m. For Salicaceae, the average canopy breadth reached 309.91 cm in one direction and 317.09 cm in the other, representing the highest values observed. Amongst Fabaceae, which exhibited the largest dimensions in terms of stem diameter, the mean diameter at breast height (DBH) was 16.45 cm, while the mean base diameter was 24.20 cm (Table 1).

For shrub species, the maximum and minimum heights were 6.66 m and 0.067 m, respectively. The average canopy breadth was 318.36 cm in one direction and 329.64 cm in the other. The base diameter of shrubs ranged from 0.500 cm to 52.00 cm, with a mean value of 329.64 cm. Maximum height and crown diameter were the highest for Salicaceae and the base diameter was the largest for Caprifoliaceae (Table 2).

### Future implication and data use

The diameter and height of tree and shrub species have a significant impact on understorey plant diversity, owing to the density effects of planted trees on species interactions and compositions. Combined with the plantation density of tree and shrub species, plant community structure may vary with diameter and height. The appropriate selection of tree and shrub species according to their diameter and height characteristics is important for the development and implementation of environmentally safe afforestation strategies. Hence, it is useful to assess the changes in species composition of plant communities in plantation forests. To enhance the dataset, we propose incorporating additional functional traits, namely wood density and leaf traits. Wood density is a key determinant of carbon storage, while leaf traits significantly influence biomass (Wan et al. 2019). Integrating these traits can improve the development of robust biomass equations for both trees and shrubs.

The databases documenting plant functional traits — such as leaf and root characteristics— have been widely utilised by ecologists and conservation biologists (e.g. the TRY database). In contrast, our study presents the first comprehensive dataset focused on dendrometric traits derived from biomass equations for tree and shrub species used in carbon sequestration projects. Such trait datasets are vital for providing information for ecological management practices (Wan and Wang 2023, Wan et al. 2024). Therefore, advances in the development of ecological indicators will benefit from datasets like ours, which are designed with specific ecological applications in mind, for example, Climate, Community and Biodiversity Standards (CCB; Wan et al. (2024)).

The Climate, Community and Biodiversity Standards (CCB) represent the assurance that a particular forest project is delivering tangible climate, community and biodiversity benefits. According to the CCB standards, it is necessary to assess the biomass of plant communities in plantation forests. Carbon sequestration projects should meet the CCB standards for climate, community, and biodiversity benefits. The accurate assessment of tree and shrub biomass is critical for reaching the CCB standards. The equation parameters of individual biomass should be provided for assessing the carbon storage of CCB projects. Our study provides an effective reference for the application of CCB in plantation forests managed by carbon sequestration projects.

## Figures and Tables

**Table 1.** Overview of dendrometric traits of biomass equations for tree species.

Famliy	Individual number	Statistics	Height (m)	Diameter at breast height (cm)	Base diameter (cm)	Canopy breadth1 (cm)	Canopy breadth2 (cm)
Cupressaceae	58	Mean	1.31	6.97	5.77	51.53	54.93
Cupressaceae	58	SD	0.79	8.00	8.27	52.26	56.76
Cupressaceae	58	Min	0.50	0.80	0.60	17.00	18.00
Cupressaceae	58	Max	4.50	25.50	39.40	320.00	350.00
Elaeagnaceae	2	Mean	2.50	1.70	4.25	154.50	156.50
Elaeagnaceae	2	SD	0.42	0.42	0.07	13.44	4.95
Elaeagnaceae	2	Min	2.20	1.40	4.20	145.00	153.00
Elaeagnaceae	2	Max	2.80	2.00	4.30	164.00	160.00
Fabaceae	187	Mean	6.40	16.45	24.20	305.05	284.98
Fabaceae	187	SD	4.15	11.29	18.26	172.46	178.93
Fabaceae	187	Min	0.40	1.00	0.90	25.00	24.00
Fabaceae	187	Max	18.00	56.00	71.00	690.00	770.00
Juglandaceae	38	Mean	1.77	5.91	9.19	134.63	117.55
Juglandaceae	38	SD	1.04	2.74	5.01	81.36	53.73
Juglandaceae	38	Min	0.40	1.50	1.00	31.00	31.00
Juglandaceae	38	Max	4.00	13.00	18.00	330.00	220.00
Pentaphylacaceae	3	Mean	0.63		3.40	59.33	65.33
Pentaphylacaceae	3	SD	0.38		0.61	35.44	27.02
Pentaphylacaceae	3	Min	0.20		3.00	35.00	45.00
Pentaphylacaceae	3	Max	0.90		4.10	100.00	96.00
Pinaceae	1191	Mean	1.66	4.56	5.07	100.24	99.33
Pinaceae	1191	SD	1.43	5.17	6.10	71.88	69.48
Pinaceae	1191	Min	0.20	0.20	0.40	13.00	10.00
Pinaceae	1191	Max	12.00	33.50	40.50	480.00	460.00
Rosaceae	191	Mean	2.81	6.70	11.61	161.22	154.58
Rosaceae	191	SD	0.95	4.42	7.83	80.97	77.45
Rosaceae	191	Min	0.60	1.10	1.90	20.00	18.00
Rosaceae	191	Max	5.70	25.00	35.90	436.00	400.00
Rutaceae	6	Mean	2.02	1.00	8.10	113.50	144.17
Rutaceae	6	SD	0.80		5.45	79.73	69.83
Rutaceae	6	Min	1.40	1.00	1.50	59.00	62.00
Rutaceae	6	Max	3.10	1.00	17.60	270.00	250.00
Salicaceae	177	Mean	7.51	11.98	13.67	309.91	317.09
Salicaceae	177	SD	4.35	8.76	9.91	185.64	197.04
Salicaceae	177	Min	0.60	0.20	0.60	14.00	17.00
Salicaceae	177	Max	18.70	40.70	48.50	759.00	774.00
Sapindaceae	25	Mean	3.14	5.21	10.54	186.12	173.52
Sapindaceae	25	SD	1.88	3.58	5.82	127.27	96.87
Sapindaceae	25	Min	1.00	1.80	2.30	45.00	65.00
Sapindaceae	25	Max	8.00	15.00	25.00	540.00	410.00
Simaroubaceae	47	Mean	2.34	5.63	10.10	140.09	148.47
Simaroubaceae	47	SD	1.59	2.93	5.48	96.26	105.25
Simaroubaceae	47	Min	0.40	2.10	2.00	32.00	30.00
Simaroubaceae	47	Max	6.50	14.10	20.00	510.00	390.00
Ulmaceae	159	Mean	4.61	8.49	11.22	205.49	197.87
Ulmaceae	159	SD	2.73	10.94	13.17	146.99	136.55
Ulmaceae	159	Min	0.70	0.20	0.90	25.00	20.00
Ulmaceae	159	Max	12.50	47.50	63.50	760.00	690.00

**Table 2.** Overview of dendrometric traits of biomass equations for shrub species.

Famliy	Bush number	Statistics	Maximum height (m)	Individual number	Base diameter (cm)	Crown diameter1 (cm)	Crown diameter2 (cm)
Anacardiaceae	118	Mean	0.79		4.07	54.46	52.20
Anacardiaceae	118	SD	0.73		3.82	40.93	42.96
Anacardiaceae	118	Min	0.10		1.00	20.00	15.00
Anacardiaceae	118	Max	3.95		18.50	270.00	285.00
Apocynaceae	1	Mean	0.80	1.00	2.70	65.00	70.00
Apocynaceae	1	SD					
Apocynaceae	1	Min	0.80	1.00	2.70	65.00	70.00
Apocynaceae	1	Max	0.80	1.00	2.70	65.00	70.00
Araliaceae	3	Mean	1.26	1.50	7.87	91.67	98.00
Araliaceae	3	SD	0.62	0.71	2.97	20.21	58.03
Araliaceae	3	Min	0.55	1.00	5.10	70.00	55.00
Araliaceae	3	Max	1.63	2.00	11.00	110.00	164.00
Berberidaceae	2	Mean	1.37	4.00	7.40	141.50	134.50
Berberidaceae	2	SD	1.01	1.41		94.05	34.65
Berberidaceae	2	Min	0.65	3.00	7.40	75.00	110.00
Berberidaceae	2	Max	2.08	5.00	7.40	208.00	159.00
Caprifoliaceae	26	Mean	1.05	11.69	15.08	150.33	133.00
Caprifoliaceae	26	SD	0.50	14.89	9.04	60.92	48.94
Caprifoliaceae	26	Min	0.31	1.00	4.90	50.00	77.00
Caprifoliaceae	26	Max	2.40	62.00	31.00	295.00	250.00
Elaeagnaceae	97	Mean	0.78	5.75	9.79	105.73	121.36
Elaeagnaceae	97	SD	0.75	5.29	10.37	74.43	75.85
Elaeagnaceae	97	Min	0.07	1.00	2.10	16.00	24.00
Elaeagnaceae	97	Max	3.00	28.00	52.00	310.00	326.00
Fabaceae	215	Mean	1.23	19.00	8.22	135.69	135.45
Fabaceae	215	SD	0.63	18.84	7.40	89.24	85.54
Fabaceae	215	Min	0.23	1.00	0.80	20.00	16.00
Fabaceae	215	Max	3.35	84.00	37.00	375.00	387.00
Grossulariaceae	6	Mean	0.77	37.33		62.00	65.17
Grossulariaceae	6	SD	0.17	18.17		24.08	26.10
Grossulariaceae	6	Min	0.57	12.00		35.00	35.00
Grossulariaceae	6	Max	0.97	57.00		95.00	96.00
Oleaceae	10	Mean	2.47	20.67	2.60	207.60	221.00
Oleaceae	10	SD	0.53	9.22		62.66	65.42
Oleaceae	10	Min	1.50	10.00	2.60	84.00	97.00
Oleaceae	10	Max	3.34	33.00	2.60	294.00	306.00
Rhamnaceae	28	Mean	1.53	2.11	8.17	131.39	132.18
Rhamnaceae	28	SD	0.52	1.22	4.83	60.37	53.61
Rhamnaceae	28	Min	0.60	1.00	2.10	40.00	35.00
Rhamnaceae	28	Max	2.90	4.00	26.00	280.00	240.00
Rosaceae	465	Mean	0.50	27.53	10.25	54.25	53.37
Rosaceae	465	SD	0.28	23.01	3.18	41.04	41.39
Rosaceae	465	Min	0.09	1.00	8.00	5.00	5.00
Rosaceae	465	Max	2.02	235.00	12.50	290.00	332.00
Salicaceae	14	Mean	3.53	17.00	10.64	329.64	318.36
Salicaceae	14	SD	1.87		8.38	198.30	207.38
Salicaceae	14	Min	0.63	17.00	0.50	65.00	69.00
Salicaceae	14	Max	6.66	17.00	23.70	603.00	724.00
Tamaricaceae	8	Mean	2.21	5.63		138.13	125.75
Tamaricaceae	8	SD	1.05	3.58		36.26	28.94
Tamaricaceae	8	Min	1.48	1.00		97.00	82.00
Tamaricaceae	8	Max	4.35	12.00		205.00	162.00
Viburnaceae	4	Mean	1.68	1.50	6.13	114.50	120.25
Viburnaceae	4	SD	0.48	0.58	2.24	31.59	19.35
Viburnaceae	4	Min	1.40	1.00	3.90	80.00	93.00
Viburnaceae	4	Max	2.39	2.00	8.10	155.00	135.00
